# *DLL1* haploinsufficiency in prenatal brain anomalies: a retrospective analysis of 6q terminal deletions

**DOI:** 10.3389/fgene.2025.1640775

**Published:** 2025-10-20

**Authors:** Tingting Ge, Xiaojuan Lin, Xinyuan Tian, Xiaoyu Song, Bingbo Zhou, Ling Hui, Xiaozhuan Wang, Zhiqiang Zhang, Chuan Zhang

**Affiliations:** ^1^ Prenatal Diagnosis Center, Gansu Provincial Maternity and Child-care Hospital, Lanzhou, China; ^2^ Key Laboratory of Maternal-Fetal Medicine and Reproductive Protection of Gansu Province, Lanzhou, China; ^3^ Center for Medical Genetics, Gansu Provincial Maternity and Child-care Hospital, Gansu Provincial Clinical Research Center for Birth Defects and Rare Diseases, Lanzhou, China; ^4^ Medical Imaging Center, Gansu Provincial Maternity and Child-care Hospital, Lanzhou, China

**Keywords:** 6q, terminal, deletion, *DLL1*, haploinsufficiency, prenatal, brain, anomalies

## Abstract

**Objective:**

6q terminal deletion is a rare genetic cause of prenatal brain anomalies. We evaluated five cases of cerebral dysplasia within a familial context for genetic diagnosis. Aims to analyze prenatal brain abnormalities from 6q terminal deletion of *DLL1* and support prenatal diagnosis and genetic counseling.

**Methods:**

A retrospective analysis was conducted on data from five families with fetal brain structural dysplasia, collected at Gansu Provincial Maternity and Child-care Hospital (Gansu Central Hospital) between January 2017 and April 2024. We applied copy number variation sequencing (CNV-Seq) and when negative, whole-exome sequencing (WES) to define genomic etiologies of prenatal brain anomalies.

**Results:**

A total of 5 fetuses were included in this study. All fetuses exhibited a cerebellar diameter smaller than expected for their gestational age, as determined by US, 4/5 cases underwent MRI. In fetuses 1–4, CNV-Seq analysis identified heterozygous deletions of 1.74 Mb, 2.88 Mb, 0.72 Mb, and 21.99 Mb at the terminal region of chromosome 6q. In fetus 5, WES successfully identified the deletion that CNV-seq had missed, likely terminal coverage drop/binning limit.

**Conclusion:**

Fetuses with reduced transverse cerebellar diameter and ventriculomegaly should be evaluated for 6q terminal deletions involving *DLL1*; combining CNV-seq with reflex WES reduces missed diagnoses and informs counseling.

## Introduction

Disorders linked to *DLL1* (OMIM: 618709) exhibit diverse neurodevelopmental symptoms, including developmental delays, intellectual disabilities, autism, attention deficits, stereotypical behaviors, and brain anomalies like hydrocephalus and dysplasia. Common symptoms also include seizures, hypotonia, joint hyperextension, ataxia, scoliosis/kyphosis, and cognitive impairment (Fischer-Zirnsak et al., 2019). Overlapping microdeletions in the 6q27 region, affecting genes like *DLL1*, *THBS2*, *PHF10*, and *ERMARD*, linked to developmental delay, intellectual disability, and brain malformations (Fischer-Zirnsak et al., 2019). During embryogenesis, *DLL1*, expressed in neural precursor cells, regulates differentiation through oscillatory Notch signaling, affecting brain development by inhibiting differentiation in adjacent cells (Fischer-Zirnsak et al., 2019; Louvi et al., 2006; Cao et al., 2019; Shimojo et al., 2011; Artavanis-Tsakonas et al., 1999). Research has shown that the Notch ligand DLL1 is crucial for developing the central nervous system, somites, and lymphocytes (Gray et al., 1999; Hrabĕ de Angelis et al., 1997; Marklund et al., 2010; Hiraoka et al., 2013; Dunwoodie et al., 1997; Okigawa et al., 2014; Mahler et al., 2010). Among these, *DLL1* is most likely intolerant to loss of function (LoF), suggesting its deficiency may contribute to neurodevelopmental disorders (Fischer-Zirnsak et al., 2019; Karczewski et al., 2020; Peddibhotla et al., 2015). Given DLL1’s role in neurodevelopment, we investigated whether 6q terminal deletions involving *DLL1* underlie prenatal brain anomalies detected by ultrasound/MRI. To delineate genomic causes, we applied CNV-seq to all cases and performed reflex WES when CNV-seq was negative.

## Methods

### Study design and setting and participants

This study employed a retrospective analysis of a consecutive series of fetuses referred to Gansu Provincial Maternity and Child-care Hospital (Gansu Central Hospital) between January 2017 and April 2024. Cases were initially identified based on prenatal ultrasound examination suggesting structural brain anomalies, and four cases were further assessed by prenatal magnetic resonance imaging (MRI). From this cohort, five cases with a confirmed genetic diagnosis of a 6q terminal deletion involving the *DLL1* gene were selected for in-depth analysis. Comprehensive clinical, genetic, and family data were collected for all included cases. Cases were excluded for incomplete clinical or imaging data, the presence of major confounding comorbidities (e.g., aneuploidy, congenital infections), or loss to follow-up.

### Sample collection

Amniocentesis was performed under sterile conditions to obtain 30 mL of amniotic fluid. Peripheral blood (2–3 mL) was collected from both parents.

### CNV-seq

CNV-seq libraries were prepared using a commercial high-throughput sequencing kit (BerryGenomics) according to the manufacturer’s protocol, including DNA end repair, adapter ligation, barcoding, and purification (Zhang et al., 2018). Libraries were pooled and sequenced on the NextSeq CN500 platform—a commercially available desktop sequencer—at a minimum depth of 1× and resolution of 100 kb. Sequencing reads were aligned to the human reference genome GRCh37 (hg19), chosen for compatibility with major clinical genomics databases. Quality control metrics including average read depth, coverage uniformity, and terminal coverage behavior were evaluated. CNV pathogenicity was assessed using DECIPHER (https://www.deciphergenomics.org/browser), OMIM (https://www.omim.org/), UCSC Genome Browser (https://www.genome.ucsc.edu/), ClinVar (https://www.ncbi.nlm.nih.gov/clinvar/), and ClinGen (https://www.clinicalgenome.org/), and interpreted according to ACMG guidelines (Riggs et al., 2020).

### Detection of WES

Whole-exome sequencing (WES) was performed on the BGISEQ-2000 platform (BGI, Shenzhen, China) using the BGI Exome Capture V1 kit (BGI, Shenzhen, China). The average sequencing depth was 200×, with 98.5% of the target regions covered at ≥30×, 99% of the target regions covered at ≥20×. Variant calling for single-nucleotide polymorphisms (SNPs) and insertions/deletions (Indels) was conducted following the GATK best practices workflow. Short-read sequencing data were aligned to the reference genome GRCh37/hg19 using BWA-MEM, followed by duplicate marking, base quality score recalibration, and variant discovery using Haplotype Caller. Final variants were filtered based on recommended quality metrics. Copy number variant (CNV) analysis from exome data was applied in this study, the resolution was> 100 kb. Detected variants were annotated and interpreted according to the ACMG guidelines (Richards et al., 2015).

### Ethics

Written informed consent was obtained from all participating families. This study received approval from the hospital’s Ethics Committee, with the approval number (No. 2024GSFY07).

## Results

### Cohort description

**FIGURE 1:**
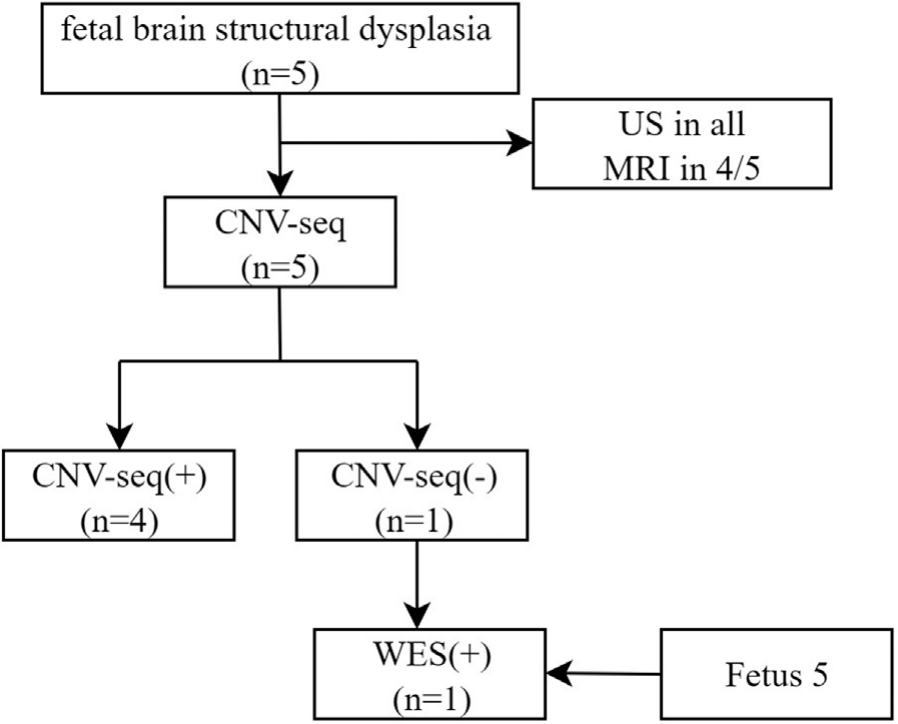
Workflow diagram.

A total of five fetuses were included in this study. The gestational age at initial diagnosis ranged from 21.56 to 27.14 (24.45 ± 1.89) weeks by ultrasound (US). Fetuses exhibited a cerebellar diameter smaller than expected for their gestational age, as determined by US and MRI (US in all, MRI in 4/5), Figure 1.

### Imaging findings

**FIGURE 2:**
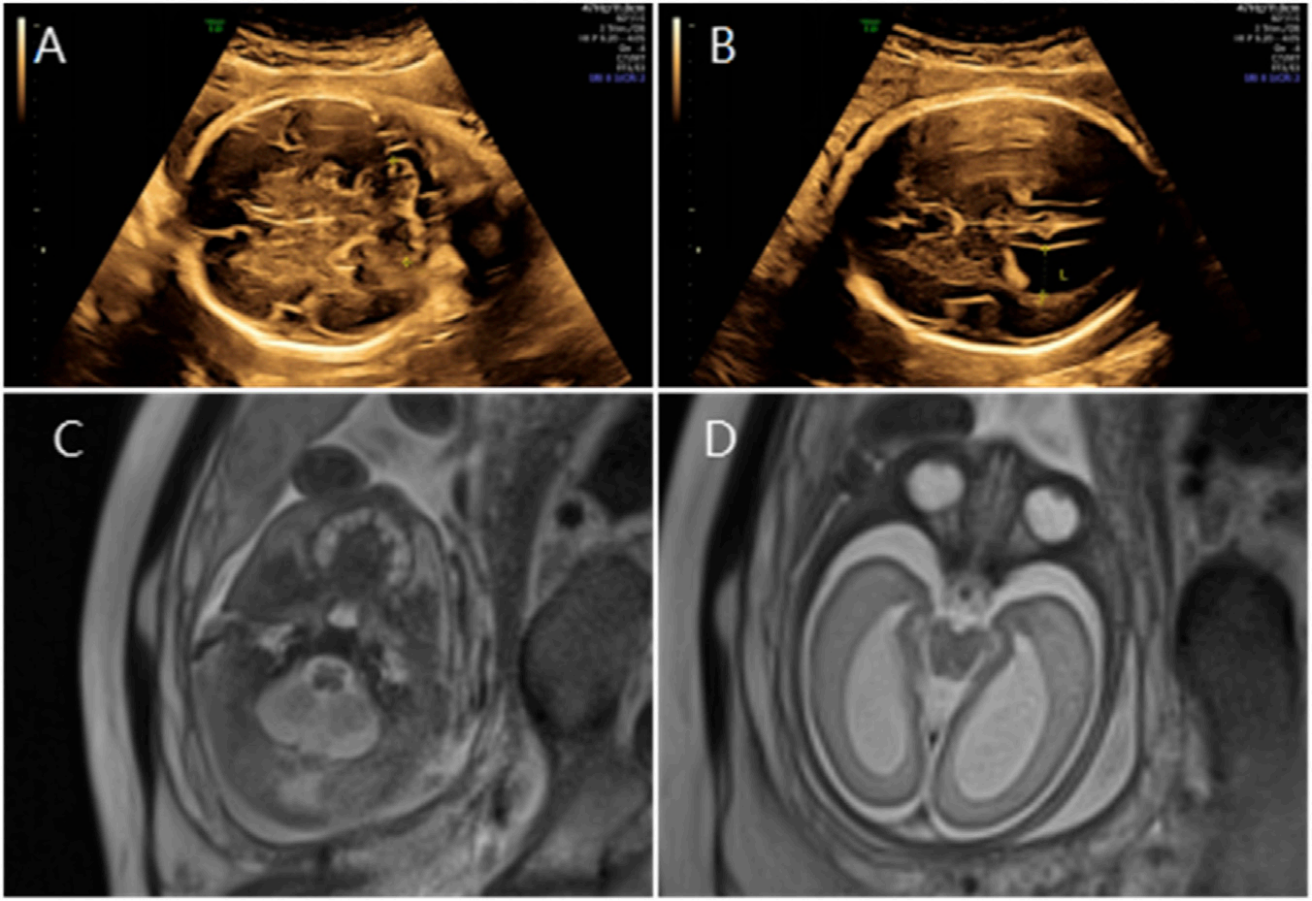
**(A)** Transverse ultrasound section showing the fetal cerebellum with a transverse cerebellar diameter of 27.8 mm (<–3.0 SD for gestational age). **(B)** Axial ultrasound view in thetransventricular plane demonstrating bilateral ventriculomegaly with atrial widths of 12.3 mm (left) and 12.5 mm (right). **(C,D)** Axial T2-weighted fetal MRI confirming reduced cerebellar size and bilateral ventriculomegaly.

**FIGURE 3:**
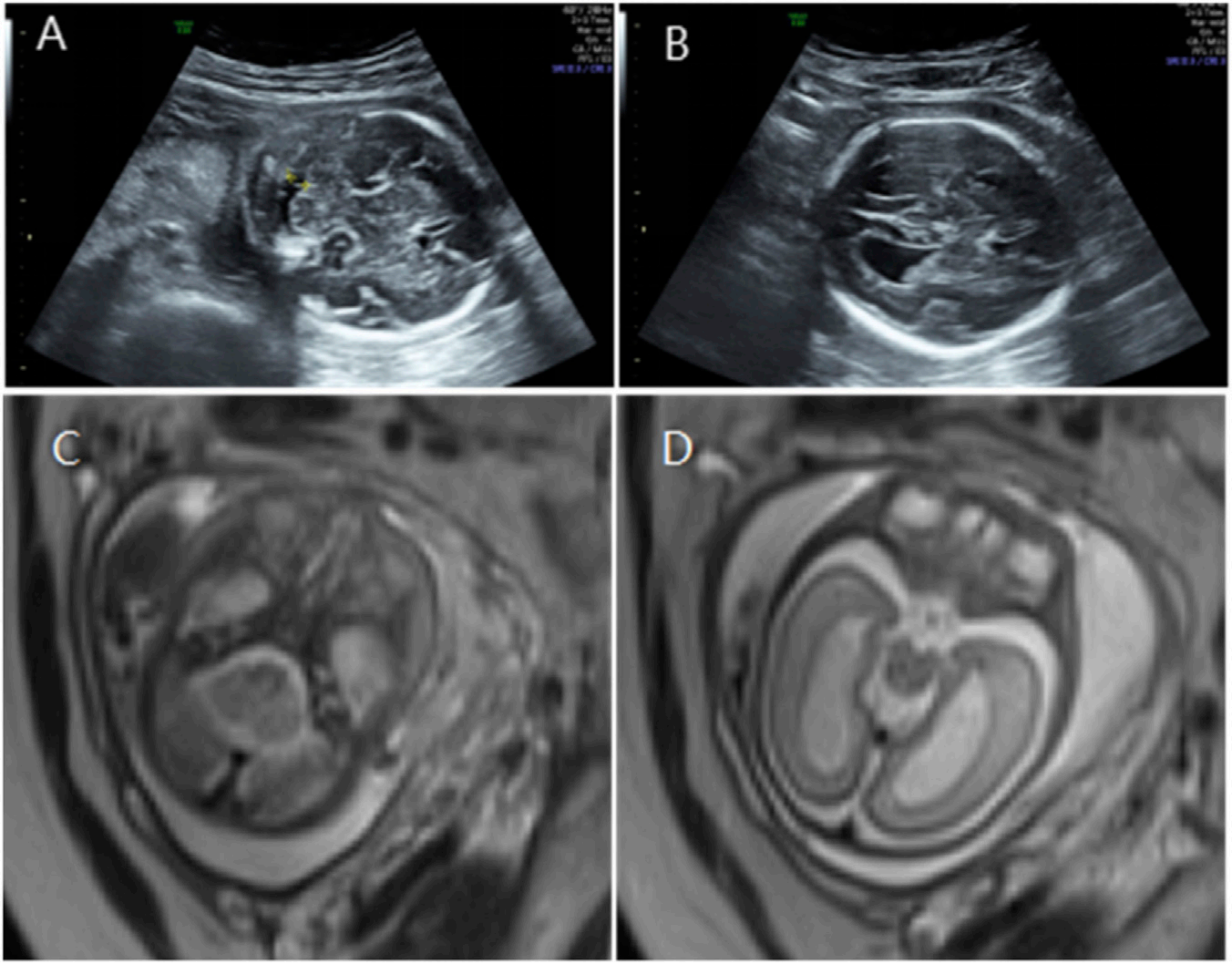
**(A)** Transverse ultrasound section showing the fetal cerebellum; transverse cerebellar diameter measured 24.8 mm (<–3.2 SD for gestational age), consistent with cerebellar hypoplasia. **(B)** Axial ultrasound view in the transventricular plane demonstrating bilateral ventriculomegaly, with atrial widths of 10.6 mm (left) and 11.2 mm (right). **(C,D)** Axial T2-weighted fetal MRI confirming cerebellar hypoplasia and bilateral ventriculomegaly.

**FIGURE 4:**
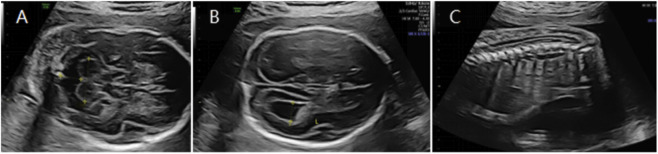
**(A)** Transverse ultrasound section showing the fetal cerebellum; transverse cerebellar diameter measured 20.2 mm (<–3.0 SD for gestational age). **(B)** Axial ultrasound view in the transventricular plane demonstrating bilateral ventriculomegaly, with atrial widths of approximately 8.9 mm (left) and 10.8 mm (right). **(C)** Sagittal ultrasound view showing a low-lying conus medullaris.

**FIGURE 5:**
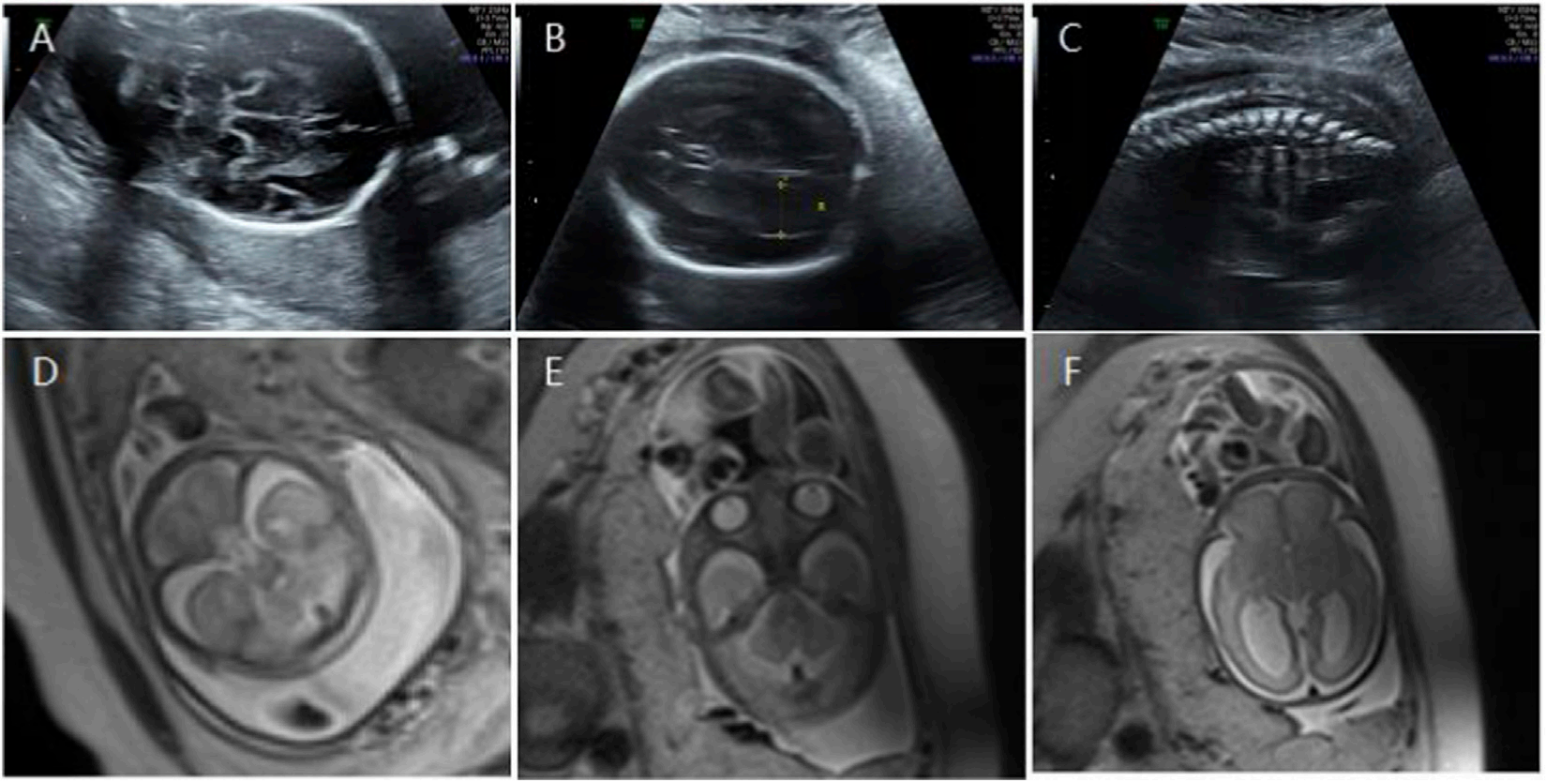
**(A)** Transverse ultrasound section showing the fetal cerebellum; transverse cerebellar diameter measured 23.4 mm (<–2.6 SD for gestational age). **(B)** Axial ultrasound view in the transventricular plane demonstrating bilateral ventriculomegaly, with atrial widths of 10.0 mm (left) and 13.6 mm (right). **(C)** Sagittal ultrasound view showing an abnormally positioned conus medullaris. **(D,E)** Axial T2-weighted fetal MRI confirming reduced cerebellar size. **(F)** Axial T2-weighted fetal MRI showing bilateral ventriculomegaly.

All fetuses exhibited a transverse cerebellar diameter smaller than expected for their gestational age, as determined by US, 4/5 cases underwent MRI (Figures 2–5). Common complications included mild lateral ventricle dilatation (5/5 cases), corpus callosum abnormalities (1/5 cases) and low positioning of the conus medullaris (2/5 cases) in a clean Table 1. Among them, fetus 4 was first found at 21 + 4 weeks of gestation with multiple system malformations, Included were fetal growth restriction, double outlet right ventricle, Nuchal fold thickened, scoliosis, hand deformities.

**TABLE 1:** Clinical data and prenatal ultrasound examination results of 5 fetuses.

Fetal	Age	Gravidity and parity	Gestational age at ultrasound diagnosis (W)	Vp left|right (mm)	TCD (mm)	CSP	CC abnormalities	Gyration abnormalities	CMD (mm)	Conus medullaris abnormalities	Additional sings	MRI
1	24	G1P0	24 + 1	13.7 | 15.1	23.4 <–3.0 SD for gestational age	Yes	Yes	Yes	8.5	No	No	Yes
2	26	G2P0	27 + 1	12.3 | 12.5	27.8 <–3.0 SD for gestational age	Yes	No	No	3.9	No	No	Yes
3	26	G2P0	25 + 5	10.6 | 11.2	24.8 <–3.2 SD for gestational age	Yes	No	No	4.9	No	No	Yes
4	28	G3P0	21 + 4	8.9 | 10.8	20.2 <–3.0 SD for gestational age	Yes	No	No	9.5	Yes	FGR, DOVR, Nuchal fold thickened, Scoliosis, Hand Deformities	No
5	33	G3P0	23 + 5	10.0 | 13.6	23.4 <–2.6 SD for gestational age	Yes	No	No	3.9	Yes	No	Yes

W, week; Vp, Ventricular width of the posterior horn; TCD, transverse cerebellar diameter; CSP, cavum septi pellucidi; CC, corpus callosum; CMD, cistern magna depth; MRI, magnetic resonance imaging; SD, standard deviation; FGR, fetal growth restriction; DOVR, double outlet right ventricle.

### Genetic findings

A heterozygous deletion was identified in all fetuses, with the deletion region located at the terminal end of chromosome 6q, encompassing the haploinsufficiency-sensitive gene *DLL1* (2A, 1 score). The total score for fragment deletion was ≥0.99, classifying it as a pathogenic variation (Tables 1 and 2). Notably, in fetus 5, CNV-seq negative, the pregnant woman opted for further WES, this analysis revealed a heterozygous deletion of 0.14 Mb in the Chr6:g.170460966-170604541 region, likely terminal coverage drop/binning limit.

**TABLE 2:** Genetic diagnosis data of 5 fetuses.

Fetal	Sample type	Detection method	CNV-seq/WES results	Deletion size (Mb)	Includes *Dll1*	Pathogenic assessments	Pregnancy outcome
1	AF	CNV-seq	Chr6:g.169180001-170920000del	1.74	Yes	Pathogenic	TOP
2	AF	CNV-seq	Chr6:g.168040000-170920000del	2.88	Yes	Pathogenic	TOP
3	AF	CNV-seq	Chr6:g.170330472-171054567del	0.72	Yes	Pathogenic	TOP
4	AF	CNV-seq	Chr6:g.149069313-171054567del	21.99	Yes	Pathogenic	TOP
5	AF	CNV-seq and WES	Chr6:g.170460966-170604541del	0.14	Yes	Pathogenic	TOP

AF, amniotic fluid; CNV-seq, copy number variation; WES, whole-exome sequencing; TOP, termination of pregnancy.

Given that fetuses with haploinsufficiency of the *DLL1* gene predominantly exhibit clinical manifestations such as intellectual disability, brain malformations, autism, epilepsy, and other related phenotypes, the parents of the five fetuses elected to terminate the pregnancy following genetic counseling.

### Genotype–phenotype

*DLL1* deletion exhibited altered brain structures, the most prevalent abnormalities were cerebellar dysplasia, increased ventricle width, and corpus callosum anomalies (observed in over 70% of cases) (Lesieur-Sebellin et al., 2022). When the 6q terminal deletion extends to 7.1 Mb, the majority of the patient’s clinical features can be attributed to the *DLL1*. Deletions exceeding 7.1 Mb result in a more severe phenotype, such as such as abnormality of the anus, either anal atresia or an ectopic anus (Engwerda et al., 2023).

## Discussion

In this study, analyze five cases of cerebral dysplasia within a familial context for genetic diagnosis. All fetuses exhibited a cerebellar diameter smaller than expected for their gestational age, as determined by US, 4/5 cases underwent MRI. Common complications included mild lateral ventricle dilatation (5/5 cases), corpus callosum abnormalities (1/5 cases) and low positioning of the conus medullaris (2/5 cases). Among them, fetus 4 was first found at 21 + 4 weeks of gestation with multiple system malformations, Included were fetal growth restriction, double outlet right ventricle, Nuchal fold thickened, scoliosis, hand deformities. We applied CNV-seq to all cases. CNV-seq identified the 6q terminal deletion in fetuses 1–4, however, in fetus 5, a deletion missed by CNV-seq was detected by WES, analysis revealed a heterozygous deletion of 0.14 Mb in the Chr6:g.170460966-170604541 region, likely terminal coverage drop/binning limit.

The study of *DLL1* and prenatal fetal phenotypes is relatively limited. Research by Sanlaville (Lesieur-Sebellin et al., 2022) explored the relationship between *DLL1* and fetal phenotypes, revealing that fetuses with a *DLL1* deletion exhibited altered brain structures. The most prevalent abnormalities were cerebellar dysplasia, increased ventricle width, and corpus callosum anomalies (observed in over 70% of cases). Gyration abnormalities were identified in 46% of the fetuses. Less common phenotypes included heterotopia cerebri, vertebral malformations, and renal abnormalities. In this study, five fetuses exhibited bilateral lateral ventricle expansion, and the cerebellar transverse diameter was smaller than expected for the gestational age, compared to typical clinical phenotypes. Two cases showed isolated chamber abnormalities.

Notably, fetus number 4 had the largest terminal deletion on chromosome 6q (21.99 Mb) and presented with the most severe phenotype. In addition to bilateral ventriculomegaly and a cerebellar transverse diameter smaller than expected for the gestational age, the condition was also associated with spinal abnormalities, including scoliosis, intrauterine growth retardation, and abnormal development of both arms. When the 6q terminal deletion extends to 7.1 Mb, the majority of the patient’s clinical features can be attributed to the *DLL1*. Deletions exceeding 7.1 Mb result in a more severe phenotype (Engwerda et al., 2023). *DLL1* haploinsufficiency plausibly explains prenatal anomalies.

CNV-Seq analysis can detect aneuploidies as well as pathogenic/likely pathogenic CNVs, significantly improving abnormality detection rates (Zhang C et al., 2018). Currently, CNV-seq achieves a resolution limit of about 100 kb. In this study, CNV-seq successfully identified 6q terminal deletions in fetuses 1–4, but failed to detect the 0.14 Mb deletion in fetus 5, a size within its theoretical detection range. This miss may be due to insufficient coverage of the 6q terminal region during sequencing. WES has been widely used clinically for diagnosing monogenic disorders (Zhang et al., 2021), can detect single-nucleotide variants, small insertions/deletions (≤20 bp), certain exon-level CNVs, and copy number changes. Fetus 5, the CNV-seq false negative was subsequently identified by WES. Thus, in prenatal cases with findings such as hydrocephalus, ventriculomegaly, or a small cerebellar diameter, WES successfully identified the deletion that CNV-seq had missed. As of May 2024, the ClinVar database lists 78 pathogenic/likely pathogenic CNVs, 25 frameshift, 7 nonsense (across 15 variants), and 4 splicing/missense mutations related to this region. Therefore, if CNV testing is negative in fetuses with these phenotypes, further analysis for single-base changes and small indels should be pursued to exclude monogenic disorders.

This study was a retrospective study with a small sample size, no postnatal phenotyping due to pregnancy terminations, imaging variability. Future work should prioritize the establishment of prospective registries, harmonized imaging metrics, capture of postnatal outcomes when pregnancies continue.

## Conclusion

Fetuses with reduced transverse cerebellar diameter, hydrocephalus, or bilateral ventriculomegaly should be evaluated for 6q terminal deletions involving DLL1. Reflex WES following negative CNV-seq reduces missed diagnoses and improves prenatal counseling.

## Data Availability

The datasets for this article are not publicly available due to concerns regarding participant/patient anonymity. Requests to access the datasets should be directed to the corresponding author, email: zhangchuan0404@163.com.
